# Early predictors of prognosis in juvenile idiopathic arthritis patients: a systematic literature review

**DOI:** 10.1186/1546-0096-12-S1-P16

**Published:** 2014-09-17

**Authors:** Pieter Van Dijkhuizen, Nico Wulffraat

**Affiliations:** 1 Paediatric immunology, Istituto Giannina Gaslini, Genova, Italy; 2 Paediatric immunology, University Medical Centre Utrecht, Wilhelmina Children's hospital, Utrecht, Netherlands

## Introduction

Juvenile idiopathic arthritis (JIA) is a heterogeneous group of disorders grouped in 7 categories. The prognosis of JIA patients varies markedly, even within these categories and ranges from mild inflammation of a single joint to involvement of multiple joints leading to damage and incapacity. The goal of treatment is to avoid these long-term sequelae and on the other hand to spare children with milder disease aggressive therapy. To achieve this and to inform patients and their parents correctly about the disease, it is essential to know the prognosis in the individual patient, preferably already at the time of diagnosis.

## Objectives

The aim of this study was to identify factors, which predict disease activity, joint damage, functional ability and quality of life (QoL) early in the course of disease.

## Methods

A systematic literature review was performed in PubMed, Embase, The Cochrane Library and PsycInfo and 3,679 articles were identified. The results were screened and critically appraised, using predefined criteria. Articles about studies that used validated outcomes, such as the Wallace criteria for disease remission, the childhood health-assessment questionnaire (CHAQ) and the juvenile arthritis damage index (JADI) and which determined predictors in the first 6 months of disease were selected.

## Results

Forty relevant articles were selected. Most of them were retrospective studies and many did not perform a multivariate analysis. The studies mostly evaluated basic demographic, clinical and laboratory predictors. Polyarticular onset predicted a worse prognosis for all outcomes, except QoL. A diagnostic delay and the systemic category predicted continuation of active disease. Notably, antinuclear antibodies (ANA) did not predict disease activity. Symmetric involvement and rheumatoid factor positivity predicted less damage. More disease activity was mainly associated with worse functional outcome. However, most predictors were not validated.

## Conclusion

Few factors were predictive of JIA prognosis. Validation of these predictors in independent cohorts was still lacking in many cases. Overall, demographic, clinical and routine laboratory values were insufficient as early predictors for the outcome in JIA. Prospective, longitudinal studies, using standardized outcome measurements, such as the Wallace criteria and the CHAQ, and evaluating a broader range of predictors, such as genetics, immunologic and imaging data, should be performed. For the outcomes joint assessment and quality of life, standardized and validated outcomes should be developed.

## Disclosure of interest

None declared.

